# Shifting fish distributions in warming sub-Arctic oceans

**DOI:** 10.1038/s41598-020-73444-y

**Published:** 2020-10-05

**Authors:** Steven E. Campana, Ragnhildur B. Stefánsdóttir, Klara Jakobsdóttir, Jón Sólmundsson

**Affiliations:** 1 Life and Environmental Science, University of Iceland, 101 Reykjavík, Iceland; 2 Marine and Freshwater Research Institute, Hafnarfjörður, Iceland

**Keywords:** Climate-change ecology, Population dynamics

## Abstract

The distributional response of marine fishes to climate warming would be expected to be very different than that of homeothermic birds and mammals, due both to more direct thermal effects on poikilothermic fish physiology and on reduced habitat fragmentation. In this study, we use a combination of linear models and graphical tools to quantify three-dimensional distribution shifts in 82 fish species caught in 5390 standardized groundfish survey tows over a 22-year time frame in the highly-productive sub-Arctic waters around Iceland. Over a 1 °C range, temperature significantly modified the distributional centroids of 72% of all fish species, but had relatively little effect on diversity. Most of the geographic shifts were to the northwest, and there was no overall tendency to move to deeper waters. A doubling of species abundance significantly influenced the distribution of 62% of species, but lacked the poleward orientation observed with temperature increases. Stenothermal species, those near their upper or lower thermal limits, and those with restricted spatial ranges were most likely to shift their distribution in response to climate warming, while deepwater species were not. A 2–3 °C warming of marine waters seems likely to produce large-scale changes in the location of many sub-Arctic fisheries.

## Introduction

Climate change is expected to disrupt biological communities in many parts of the world, with both direct and indirect effects on distribution, growth and mortality. In the terrestrial environment, the species most likely to be negatively affected will be those with inflexible life history traits such as slow reproductive rates, specialised habitat and dietary requirements, and narrow physiological tolerances^1,2^. Range loss, limited dispersal capabilities and barriers to dispersion are expected to be particularly strong influences for terrestrial mammals, leading to increased risk of extinction in some areas^3,4^. Yet the response of poikilothermic fishes to warming would be expected to be very different than that of homeothermic birds and mammals, which account for the vast majority of existing animal studies on climate effects. Temperature has a pronounced and immediate effect on fish growth and productivity compared to most homeotherms^5^. Conversely, dispersal barriers and habitat fragmentation may be less of an issue for marine fishes than for organisms in the terrestrial environment, especially in the open ocean^6^. As a result, range expansions and contractions in the marine environment would be expected to be a regular part of an ongoing process in a changing environment, even though migration pathways and spawning grounds are far less easily changed^7^.

Fish species near the limits of their thermal distribution would appear to be obvious candidates for range shifts with rising temperatures, and many freshwater fish species show the expected range shifts in response to changes in temperature and rainfall^8,9^. In the marine environment however, there are surprisingly few large-scale, multispecies predictors of distributional shifts that are not confounded with changes in abundance or fishing. In an analysis of North Sea fish species surveyed over a 25-year period, the only species that shifted their distribution deeper or to the north were shallow, warmwater species or those with range margins in the North Sea; fishing appeared to have little effect^10,11^. A second study subsequently reported that North Sea cod shifted northwards and to deeper waters due to warming after 1913, but that it was fishing-related depletion that shifted them to the east^12^. In a third study of North Sea cod, it was reported that both earlier studies erred in ignoring different cod population sensitivities to temperature, and that the apparent shift to the north was actually a reflection of different population growth rates^7^. Elsewhere, temperature has variously been reported to have a small effect on area occupied due to confounding changes in population abundance^13^, a mixed effect due to both temperature and fishing-induced changes in abundance^14^, and a strong driver of distributional shifts at some ontogenetic stages but not others^15^. It has also been argued that thermal extremes alter distribution not through behavioural means, but through their effect on oxygen limitation and the subsequent reduction in growth and abundance^16^. Disentangling the effects of rising temperatures and changing abundance is by no means straight forward, implying that climate-induced distribution shifts may occur because of active movement of individuals, or geographic shifts in recruitment or survival rates, or both.

It seems inescapable that temperature extremes ultimately limit the distributional range of marine fishes, whether through behavioural or physiological means^17^. However, the location of many spawning grounds and migration pathways is evolutionarily stable, thus constraining the range of re-distributions that are possible as the environment changes^18^. Thus it remains unclear if moderate and gradual temperature increases in the ocean will shift overall distribution independent of natural or fishing-related changes in population abundance. In part, the conflicting conclusions to date may stem from the very different indices of distributional change that have been used. Range extensions based on presence/absence data have been reported to be more sensitive to distributional shifts than abundance-based data, but are also more sensitive to spurious effects and search effort; intensified search is more likely to detect rare but endemic species^19^. Abundance-weighted distributional data would seem to be the best representation of the entire population, but it is well documented that increased population abundance will expand the range that is occupied, even if the increased abundance is due to an increase in year-class strength or reduced fishing effort^20^. Multispecies indicators such as community assemblages and diversity indices have been used in some studies of geographic change^11,21^, but the results were not easily transferable to other regions. In contrast, the few studies which have used functional measures of climate-induced geographic shifts have provided promising results. For example, Frainer et al.^22^ monitored functional traits in 52 fish species in the Barents Sea over 8 year of research surveys, and reported that small bottom benthivores were being replaced by large motile generalist species like cod and haddock as waters warmed. In an analogous approach, Dulvy et al.^11^ attempted to classify 28 North Sea species into assemblages based in part on their thermal preferences. In both of these studies, the intent was to infer distributional shifts based on changes in the characteristics of the species or communities, rather than the actual species themselves.

Although some previous studies of warming-induced distributional shifts in marine fishes have successfully confirmed the presence of changes, few have quantified the magnitude or orientation of the shift, nor predicted future responses. In this study, we take advantage of standardized groundfish abundance surveys of up to 200 fish species conducted in highly-productive sub-Arctic waters around Iceland over a 22-year time frame to differentiate distributional shifts due to warming from those due to abundance changes. We start by using a range of quantitative methods to identify those species which have exhibited geographic or bathymetric changes, both through time and with warming. We then develop some graphical and quantitative tools for assessing both the magnitude and orientation of the distribution shifts, and the ecological traits of the most sensitive species. We conclude by predicting the distributional response of the Icelandic fish community to future warming in a simple spatial model, thereby highlighting the diverse range of responses that can be expected in sub-Arctic marine environments.

## Results

### Ocean temperature

**Figure 1:**
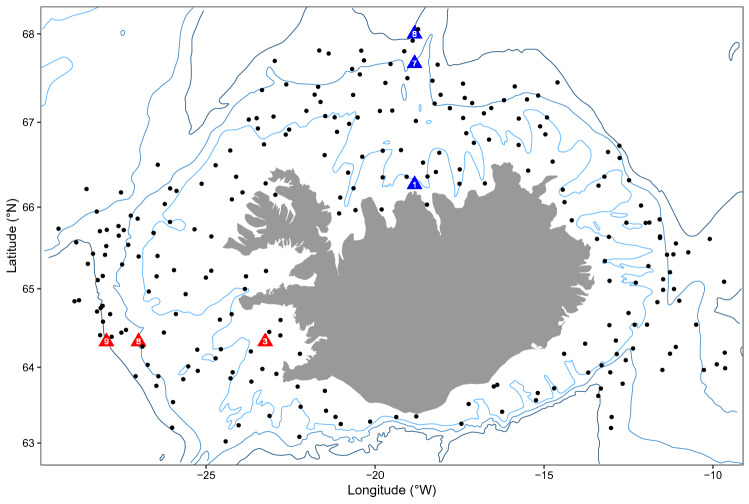
Map of 245 autumn groundfish survey core stations (black dots) sampled around Iceland every October between 1996 and 2018 (except 2011). Bottom-temperature profiles were collected independently at hydrographic stations FX3, FX8 and FX9 (red triangles) and SI1, SI7 and SI8 (blue triangles). Depth contours are 200, 500 and 1000 m.

The independent bottom water temperature estimates all showed variable but increasing autumn temperatures from 1996 to 2010, followed by a decline through to 2018 in the mid-depth offshore stations and the survey, but less of a decline in the inshore or deep offshore stations (Fig. 1, 2a). Both deep offshore stations warmed by ~ 0.2 °C over the time series. The ~ 1 °C temperature range evident in the southwestern mid-depth offshore FX8 autumn hydrographic series was larger than that of the less variable northern mid-depth offshore SI7 hydrographic station, but neither of the mid-depth offshore stations showed any net warming over the 22-year period. The shallow inshore stations both warmed considerably (1.5–2 °C) over the time series. Although no net increase was apparent in either of the mid-depth offshore hydrographic station autumn time series, 0.25–0.40 °C increases were observed in both the winter and spring measurement time series at both mid-depth hydrographic stations.

**Figure 2:**
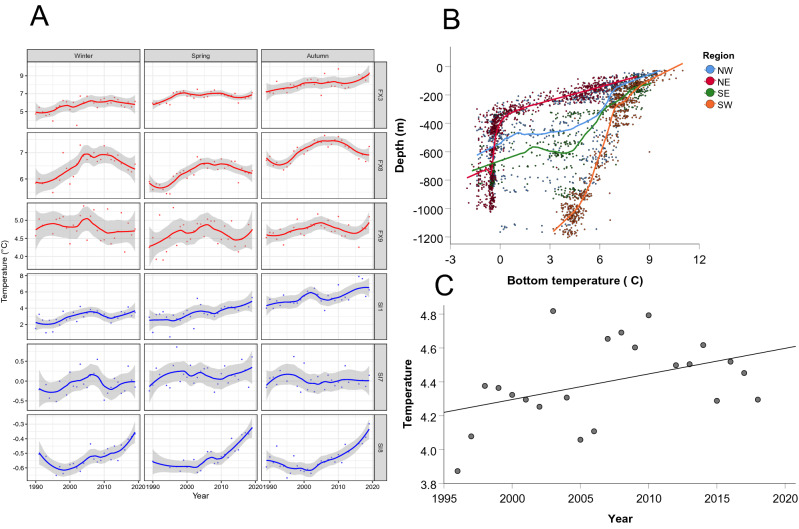
Bottom-temperatures from hydrographic stations (**A**: left panel) and from the autumn groundfish survey (**B**: top right and **C**: bottom right panel). (**A**) Near-bottom temperatures at hydrographic stations FX3 and SI1 (~ 70 m), FX8 and SI7 (~ 400 m), and FX9 and SI8 (~ 1000 m) between 1996 and 2018 are shown for winter (January–March), spring (May–June) and autumn (October–December). A geometric smooth has been fitted to the annual means. Note different scales on the y-axes. (**B**) Mean depth-temperature profiles for each region based on bottom temperatures from the autumn survey. Fitted lines are loess regressions. (**C**) The mean annual environmental temperature time series estimated from a GLM of the autumn survey bottom temperature measurements (see text for details). The linear regression fitted to the annual means is intended only to show the overall rate of warming through the time series.

The mean environmental temperature from the autumn survey (*Temp*_*e*_) lay midway between that of the two mid-depth offshore hydrographic stations, over a similar ~ 1 °C temperature range. Based on a fitted regression to the environmental temperature time series, there was a net warming of 0.33 °C across the survey area over the 22-year time period, although the increase was certainly not linear (Fig. 2c). There were large differences in both the temperature range and temperature-at-depth among some of the regions (Fig. 2b). Bottom water temperatures at 200 m depth were 3–4 °C warmer in the south (SW = 7.3 °C; SE = 8.2 °C) than in the north (NW = 5.4 °C; NE = 4.5 °C), and 4–5 °C warmer at a depth of 500 m (NW = 1.4 °C; NE = −0.5 °C; SW = 6.6 °C; SE = 3.9 °C). Although most stations deeper than 500 m in the NE were < 0 °C, all of the stations at that depth in the SW were > 3 °C.

### Survey catches

A total of 5390 tows were fished over the 22-year time series. Station depths ranged from 26 to 1203 m (mean of 377 m) while bottom temperatures ranged between − 2 and 11 °C (mean of 4.85 °C). Of the 7,246,474 fish representing 200 species that were caught, 82 species were caught during at least 19 of the survey years, and thus are the primary subject of this analysis (Suppl. Table 1). The mean standardized annual abundance of these 82 species ranged between 1 and 27,513 fish, with an overall mean across species of 4096; 70% of the species had a mean standardized annual abundance of less than 100.

There was no clear identifier of species that were rare, and thus caught sporadically, versus those that were newly immigrated to the survey area in response to the warming environment. Nor was it possible to exclude the possibility that certain rare species were incorrectly identified in some survey years. However, there were 6 species that were never caught prior to 1998, yet appeared in increasing numbers in subsequent years (Table 1). All of these species were caught in at least 8 survey years, and as many as 19. However, three of the species were deepwater (> 800 m), and thus unlikely to have been subject to temperature increases of > 0.2 °C. Of the remaining three species, Atlantic mackerel (*Scomber scombrus*), blackbelly rosefish (*Helicolenus dactylopterus*) and hollowsnout grenadier (*Coelorinchus caelorhincus*) were all warmwater (TB > 2) and stenothermal (Steno < 3.4), thus making warming temperatures a likely explanation for their recent arrival to the Icelandic fish community. There were no species that were originally present in the survey, but absent after 2015.

**Table 1:** Characteristics of fish species which first appeared in the autumn survey after 1997 and which increased in abundance in subsequent years.

Species	First year of appearance	Number of years in survey	Latitude (°N)	Longitude (°W)	Depth (m)	Temperature (°C)	Steno index	Thermal bias
*Scomber scombrus*	2005	12	64.32	20.12	139	7.9	3.2	2.5
*Helicolenus dactylopterus*	1998	19	63.43	21.25	301	7.7	1.9	2.1
*Coelorinchus caelorhincus*	2007	11	63.35	24.37	448	7.3	3.4	2.0
*Trachipterus arcticus*	2005	8	64.80	27.69	837	5.1	3.2	− 1.0
*Holtbyrnia anomala*	1999	15	64.65	28.00	1048	4.1	2.4	− 1.5
*Platytroctidae* spp.	1999	13	64.87	26.69	940	4.4	2.3	− 1.1

Values shown are all means.

### Fish thermal habitat

**Figure 3:**
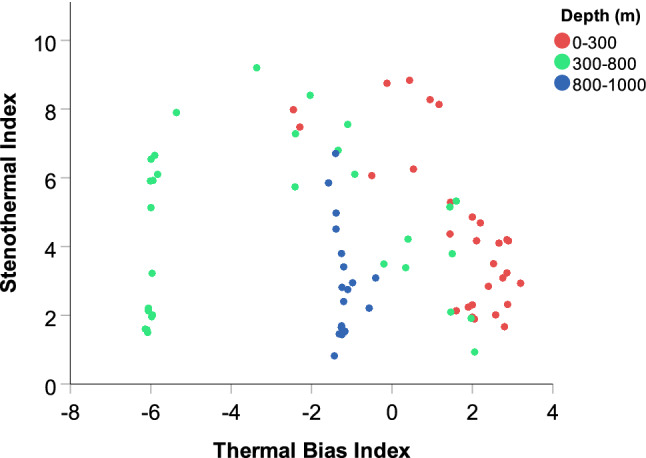
Temperature affinity indices of 82 fish species as a function of mean occupied depth (m). The Thermal Bias (TB) index of an individual species may be towards waters that are warmer (> 0) or colder (< 0) than that of the overall environment (i.e. a warm-water (> 0), cool-water (− 3 to 0) or cold-water (− 7 to − 3) species). The Stenothermal Index indicates the range of temperatures which are occupied: narrow (stenothermal) or broad (eurythermal).

Fish were caught over the entire temperature range of the survey (− 2 to 11 °C). However, not all temperatures were equally represented by fish species. For the 82 species caught in at least 19 years, the mean thermal bias index (TB) was − 0.9 (range of − 6.1 to 3.2). Most species (n = 49) were associated with waters cooler than the overall environmental mean temperature of 4.85 °C, while the remaining 33 species were in waters warmer than the environmental mean. The mean Steno index of 4.0 (range of 0.8–9.2 °C) indicated that most species were relatively stenothermal, and thus intolerant of a broad range of temperatures. Almost all shallow water species (< 300 m) were warmwater (TB > 0), while the mid-depth species (300–800 m) were mainly coldwater; perhaps because of the presence of so many deepsea species in relatively warm southerly waters, all of the deepwater species were classified as coolwater, not coldwater (Fig. 3). Both shallow and deepwater species tended to be relatively stenothermal (Steno < 5); the most eurythermal species were the species from the mid-depths (300–800 m).

The thermal habitat of most fish species could be predicted reasonably well based solely on thermal affinities. A GLM of temperature-at-capture with *Species* as the only factor explained much of the temperature variance (*P* < 0.001, r^2^ = 0.51) (Suppl. Table 2). Addition of *Year* as a covariate did not improve the explained variance, but all terms were significant (GLM, *P* < 0.001, r^2^ = 0.51) (Suppl. Table 2b). The slope of the *Year* term was statistically significant at 0.0026 (SE = 0.00014), but there is probably limited biological significance of residency in waters which are only 0.06 °C warmer by the end of the time series. Inclusion of an interaction term between *Year* and *Species* was significant, but did not improve model fit. A model which replaced the *Year* term with the annual environmental temperature (*Temp*_*e*_) and *Depth* (as covariates) produced a better overall model fit (GLM, *P* < 0.001, r^2^ = 0.62) (Suppl. Table 2). The parameter estimates for *Temp*_*e*_ (0.718 ± 0.003) and *Depth* (− 0.007 ± 0.00001) suggested that many fish species tolerated an increased temperature as the environmental temperature increased or as they moved to shallower water. Again, the interaction term between *Species* and *Temp*_*e*_ was significant, but did not improve overall model fit.

The influence of changing fish abundance on thermal habitat was tested by including standardized annual abundance (*SA*_*y*_) as a covariate in the above GLM, both as a main effect and with slopes nested within species (*P* < 0.001, r^2^ = 0.62) (Suppl. Table 2). All terms were significant, although *SA*_*y*_ as a main effect was much less significant (*P* = 0.05) when the interaction term with species was present. It was easiest to see the influence of *SA*_*y*_ on thermal habitat by regressing the standardized residuals of a GLM model with no abundance term on *SA*_*y*_. A total of 19 of the 82 species had significant relationships between the GLM residuals and standardized annual abundance (LM, *P* < 0.05), of which 13 of the 19 species had negative slopes (indicating a reduced sensitivity to increasing temperature with increased abundance). However, only 9 of the 19 species had mean annual abundances of more than 100 fish. Therefore, if there was an effect of within-species abundance on their thermal habitat, it was not strong.

### Temporal shifts in depth distribution

There was no evidence of strong shifts in depth distribution across the time series of the survey. Of the 82 species, 22 species showed significant inter-annual trends in depth: 10 of these were negative (moving shallower) and 12 were positive (moving deeper). There was no significant relationship between the regression slope parameter of these 22 species and either the Steno or the TB indices (LM, *P* = 0.10, 21 *df*).

A GLM of fish depth at capture with *Species* as a factor and *Year* as a covariate resulted in a *Year* parameter of − 0.287 ± 0.008 (GLM, *P* < 0.001, r^2^ = 0.86), suggesting that there was a net shift to waters that were about 6 m shallower across the 22 survey years (Suppl. Table 3). The interaction term between *Species* and *Year* was significant, but did not appreciably improve model fit.

### Shifts in spatial distribution

**Figure 4:**
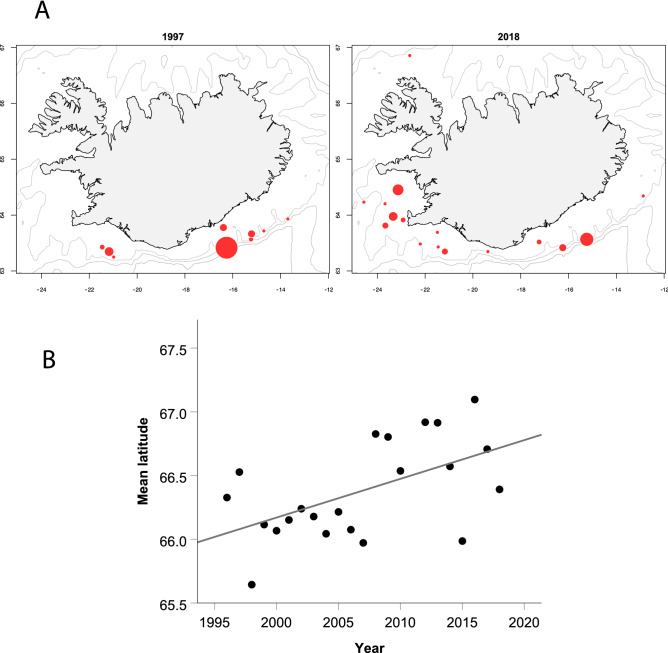
Long-term shifts in species distribution were often difficult to see in abundance-weighted maps, but were visible in species such as the grey gurnard, *Eutrigla gurnardus*, between 1997 and 2018 in the autumn survey. (**A**) Survey catch locations in early and recent years, where symbol size is proportional to catch number, exemplified the long-term trend. All years are shown in Suppl. Figure 1; (**B**) time series of mean weighted latitude (°N), fitted with a linear regression, showing northwards shift across years.

Spatial shifts in species distribution across years were often difficult to detect in distributional maps, due to routine inter-annual variance associated with the groundfish survey (Suppl. Figure 1). In addition, changes in population abundance often confounded any distributional patterns that might have been present. In species such as the grey gurnard (*Eutrigla gurnardus*), long-term distributional shifts were visible as a northwestwards shift in abundance over the 22-year survey period (Fig. 4a). A time series of the latitudinal centre of mass confirmed a significant northwards shift in distribution at a mean annual rate of 0.014° latitude·year^−1^ (LM, *P* < 0.001, 21 *df*, r^2^ = 0.64) (Fig. 4b). For most species however, the analyses reported below provided more robust indicators of distribution shifts.

**Figure 5:**
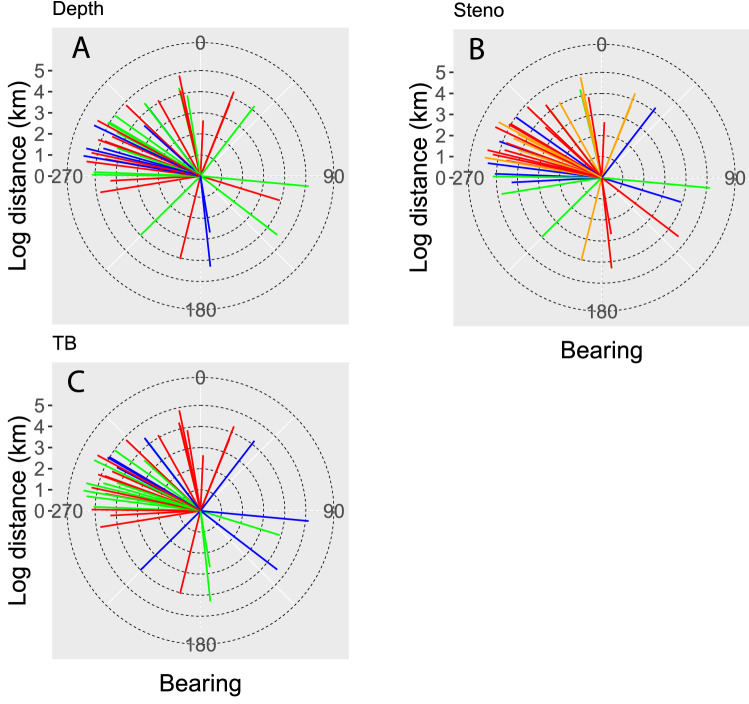
Rose plots showing estimated net movement in bearing (direction) and distance (log km) for 41 species with a significant trend in latitude or longitude over the 22-year duration of the autumn groundfish survey. (**A**) Colours indicate depth: red = shallow (< 300 m), green = mid-depth (300–800 m) and blue = deepwater (> 800 m) species. (**B**) Colours indicate Steno Index: red = most stenothermal (0 to 2.5); orange = 2.5 to 5.0; green = 5.0 to 7.5; blue = most eurythermal (7.5–9.5). (**C**) Colours indicate TB Index: blue = coldwater (− 7 to − 3); green = coolwater (− 3 to 0); red = warmwater (0 to 4).

Rose plots better showed the estimated direction and distance of net movement of each species. Of the 82 species analyzed, 41 of the species showed significant inter-annual trends in either latitude or longitude across 22 years. Almost all 41 species showed directed movement to the north and northwest (Fig. 5). This pattern was especially evident in shallow and mid-depth species, stenothermal species, and warmwater species. Deepwater species were most likely to move in any direction.

A multivariate GLM of location at capture (latitude, longitude and depth) with *Species* as a factor, and *Year* and *SAy* (species’ standardized annual abundance) as covariates, allowed the effects of time trends and species abundance trends on species’ distributions to be evaluated (GLM, *P* < 0.001) (Suppl. Table 4). Inclusion of interaction terms between each of the covariates and species allowed for different trends in each species. The model was highly significant (GLM, *P* < 0.001), explaining 26%, 34% and 86% of the variance for latitude, longitude and depth, respectively (Suppl. Table 4b). Many of the interaction terms were significant, indicating the presence of species-specific trends. However, overall patterns were evident by examining the same model without interaction terms (Suppl. Table 4a). The positive *Year* slope parameters for latitude (0.011 ± 0.001), longitude (0.035 ± 0.001) and depth (0.139 ± 0.005) indicated that there was an overall distributional shift towards the northwest and deeper waters through the survey time series. Increasing abundance produced a similar northwestern shift (latitude: 0.013 ± 0.001; longitude: 0.327 ± 0.002), but into shallower waters (− 13.6 ± 0.1).

### Temperature and abundance effects on distribution

There is no logical reason why the survey year should influence species’ distribution, except through correlation with a direct effect such as temperature or species’ annual abundance. This hypothesis was tested through use of a model similar to that described above, but with the *Year* term replaced by environmental temperature (*Temp*_*e*_). Thus the model was a multivariate GLM of location at capture (latitude, longitude and depth) with *Species* as a factor, and *Temp*_*e*_ and *SA*_*y*_ as covariates, plus the interaction terms between each of the covariates and species. The resulting model was highly significant (GLM, *P* < 0.001), explaining 25%, 34% and 86% of the variance for latitude, longitude and depth, respectively (Suppl. Table 5). Most of the interaction terms were significant, indicating the presence of species-specific trends in distribution with both temperature and abundance.

Environmental temperature as a covariate in the above model was a significant predictor of the latitude, longitude and depth of almost all species (Suppl. Table 5). As a main effect, the *Temp*_*e*_ parameter indicated an overall increase in latitude across species of 0.267 ± 0.003° for every 1° increase in temperature. When combined with the interaction term, which allowed for species-specific movements, 70% of the 82 species were predicted to move northwards as temperatures increased. A *Temp*_*e*_ parameter of 6.78° ± 0.29° for longitude indicated an overall shift to the west with warming. Again, most *Species* by *Temp*_*e*_ interaction terms were significant, indicating species-specific shifts in longitude with temperature. A total of 60% of all species would be predicted to move westward with warming water temperature. The *Depth* parameter, plus many of the interaction terms, were also significant, predicting an overall movement to shallower waters of 29.5 ± 6.4 m for every 1 °C increase in temperature; 60% of the species were predicted to move into shallower water.

Standardized species-specific annual abundance (*SA*_*y*_) was a significant term in the GLM, both as a covariate and in interaction with *Species* (Suppl. Table 5). However, abundance did not provide a consistent change in latitude, longitude and depth across species. With a doubling of *SA*_*y*_, 52% of species would be predicted to move northwards, 49% would move westwards, and 36% would move deeper.

### Spatially-explicit predictions of distribution shifts with warming and abundance

**Figure 6:**
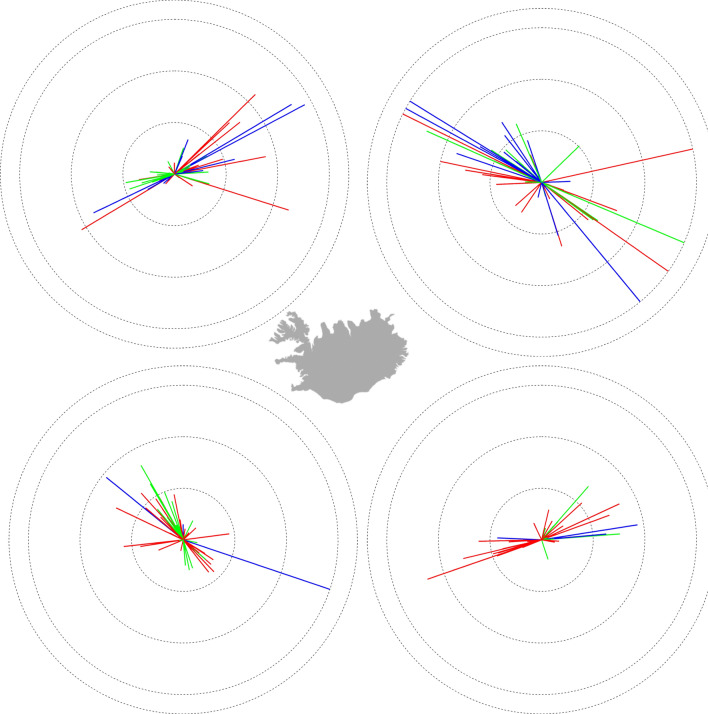
Regional rose plots showing estimated net movement in bearing (direction) and distance (km) for 59 species with a significant trend in latitude or longitude over the 22-year duration of the autumn groundfish survey. Each vector represents a species colour-coded by their Thermal Bias Index: blue = coldwater; green = coolwater; red = warmwater. Circular contours represent 50-km distances, constrained to a maximum of 150 km.

More detailed predictions of distribution shifts in response to temperature increases were possible with a multivariate GLM of the location of each species with *Region* as a factor, *Temp*_*e*_ and *SA*_*y*_ as covariates, and interaction terms between each of the covariates and *Region*. Thus the model allowed for species-specific shifts in latitude, longitude and depth within each region, without the geographical land mass barriers that might be imposed by a non-regional model. The model was fitted separately for each species (Suppl. Table 6). All models were highly significant (GLM, *P* < 0.001), with 72% of the 82 species showing significant parameter estimates for *Temp*_*e*_ as either a main effect or in interaction with *Region*. Rose plots for these 59 species showed clear distribution shifts in response to a 1 °C increase in temperature (Fig. 6). However, the distribution shifts differed among regions, whereby most species (except those living in deepwater) moved either offshore or along-shelf; only one species moved closer to shore by more than 30 km. Of the 20 species predicted to shift distributions by more than 100 km, 45% were warmwater. Although more coldwater species tended to move long distances in the north, coldwater species were also more prevalent in the north. A similar pattern was observed in the south, where warmwater species were more likely to move long distances to the south, but were also more prevalent in the south. The mean predicted distribution shift over all regions was 38 km (range of 1–326 km), with 7% of the species predicted to shift their centre of mass by more than 100 km.

Although both temperature and abundance were found to influence regional species distribution in the GLM, logic suggests that temperature increases would tend to shift populations northwards, while abundance increases would be unbiased with respect to direction. Our analysis indicated that the mean predicted increase in latitude with a 1 °C increase in temperature for all species and regions with a significant *Temp*_*e*_ parameter estimate would be a 0.074° ± 0.031° shift to the north (~ 8.2 km) in conjunction with a shallowing of 126 ± 75 m. Of the 100 species/region combinations which were significant, 62% would be predicted to move northwards. The predicted latitude shifts did not differ significantly among the four regions (*P* > 0.08), but the two western regions accounted for most of the northward-moving species. The northward prediction differed significantly from a zero net shift (*P* < 0.05), while the depth prediction did not (*P* > 0.10).

Species abundance (*SA*_*y*_) was a significant effect in the GLM for 51 of the 82 species (Suppl. Table 6). The mean predicted increase in latitude with a doubling of annual species abundance was 0.036° ± 0.025°, a mean which did not differ significantly from zero (*P* > 0.10). Overall, 55% of the regional species would be predicted to move northwards, a percentage which does not differ significantly from a random orientation (Chi-squared test, *P* > 0.10). The difference in regional latitude shifts was not significant (*P* > 0.10), but virtually all of the predicted northwards movement took place in the northeast region. Most species (61%) were predicted to move to waters which were 15 ± 8 m shallower.

A matched pair comparison for each species/region combination allowed an evaluation of the relative contribution of temperature and abundance to distribution shifts wherever both parameters were statistically significant. Of the 40 species/region combinations, 55% would be predicted to move northwards more after a 1 °C increase in temperature than after a doubling of species abundance. The mean predicted latitudinal shift was 3.4 times greater for the temperature effect than the abundance effect.

### Functional predictors of warming-induced distribution shifts

**Figure 7:**
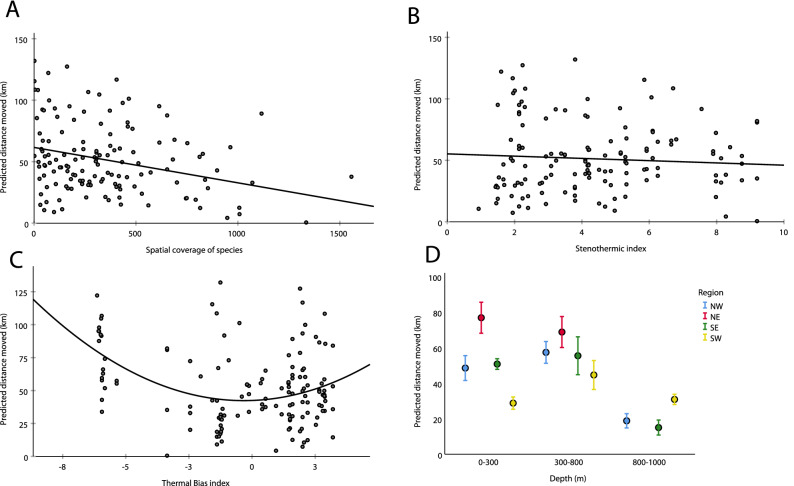
Predicted distance moved by each species in four regions around Iceland as a function of species’ ecological characteristics. The 67 species shown were those that showed significant regional temperature responses in the spatially-explicit model described in the text. (**A**) spatial coverage (number of stations occupied); (**B**) Steno index; (**C**) TB index; (**D**) depth.

The statistical models described earlier were essential to identify those species sensitive to warming-induced distribution shifts. However, it is more interesting to identify the overall physiological and ecological characteristics that made those species sensitive. A GLM of the predicted distance shifted in response to a 1° increase in water temperature included those biological variables with the potential to influence distribution: *Depth* (0–300, 300–800 and 800 + m), the *Steno* and *TB* indices, regional species’ coverage (*Area*_*sr*_) and abundance (*A*_*r*_), all in interaction with *Region* (Suppl. Table 7). All factors except *A*_*r*_ were significant, although the significant effect of *Steno* was through its interaction with *Region*. Overall, the strongest influences on the predicted distance shift were *Region*, *Steno*, *Depth* and *Area*_*sr*_. Similar results were obtained if the analysis was restricted only to deepwater or shallow species, or if the predicted latitudinal or depth shift was used as the dependent variable rather than distance. The marginal effects of each of these biological characteristics is shown in Fig. 7. Distance shifted was considerably reduced in deepwater species and in species with broad spatial coverage. Stenothermal species shifted greater distances than eurythermal species. However, the pattern with *TB* was particularly interesting: coldwater and warmwater species were more likely to shift distributions than those species residing in intermediate temperatures.

## Discussion

There is little debate surrounding the principle that climate warming should induce geographic re-distribution in marine fishes; what has been lacking to this point are quantitative predictions of the magnitude and direction of the re-distribution in a complex ecosystem. Thus our finding that climate warming will result in a net movement of 72% of the fish species to avoid warmer waters was to be expected, but not so the nonlinearity of the process, the very different sensitivities of the species, and the relatively small effects of depth shifts and fish abundance. Despite a 22-year time interval over which air temperatures over Iceland have increased by ~ 1 °C^23^ bottom water temperatures around Iceland have only increased by an average of 0.33 °C overall, and not at all in some regions. Thus the magnitude of ocean warming has been relatively small over a relatively long time period. Nevertheless, it appears that fish re-distribution has largely kept pace with the changing environment in a slow and ongoing process, reversing itself as marine waters temporarily cooled. These findings are at apparent odds with studies in the North Sea, Barents Sea and Northwest Atlantic, which reported fairly dramatic and large-scale shifts in species distribution and composition over a similar time frame^10,13,22^. However, the differences among studies may be more apparent than real. Although our median geographic re-distribution rate of 38 km °C appears modest, it is consistent with rates reported in the North Sea^11^ and obscures very large differences among species with varying ecological attributes. Thus the temporal rate of change was small around Iceland, presumably due to the oceanographic mixing discussed later, but the temperature-driven rate of change was consistent with other studies.

In Icelandic waters, Valdimarsson et al.^24^ reported 31 examples of species introduction or abundance increase since 1996 which they attributed to climate warming, but most of the examples were anecdotal, and no quantitative analyses were reported. Few of the species identified in Valdimarsson et al.^24^ could be corroborated as being climate-sensitive in our analysis. In contrast to Valdimarsson et al.^24^, another study^21^ reported no net change in Icelandic fish species diversity between 1996 and 2007, although there was a shift towards more warm-water fish species during a warming period, and different diversity trends between north and south. The modest increase in diversity apparent in our study is consistent with that of Stefansdottir et al^21^. However, we suggest that a focus on diversity ignores the more substantial effect of warming on geographic re-distribution of the existing species complex. We further argue that recruitment-driven or fishing-induced changes in abundance will inevitably change the range of a species^20^, and thus must be statistically disentangled from range shifts due only to climate. To our knowledge, the current study of fish assemblages in Icelandic sub-Arctic waters is the first to disentangle the confounding effects of climate and abundance on the lateral and depth distribution of fish in a large marine ecosystem, and then provide quantitative predictions of future climate effects.

Geographic shifts of Icelandic marine fish species were large-scale, but certainly not universal, and not always in a pole-ward direction; our results made clear that east–west shifts were sometimes optimal for reaching preferred temperature regimes, especially in southern regions where northward movement by fish was geographically impossible. Coldwater species were most likely to shift their distributions in the cold northern regions, while warmwater species were more likely to shift in warmer southern regions. Although most deepwater species lived in cool (not cold) water, deepwater species shifted their locations relatively little, and even then, were less likely to shift in a directed orientation. Such a result is consistent with the very small temperature variability present at great depths (Fig. 2). Stenothermal species were more likely to shift distributions than eurythermal species, presumably because they would be most sensitive to any temperature increases^25^. Perhaps most interesting was the finding that species with restricted distributional ranges were most likely to shift as temperatures warmed, suggesting either that they shifted to remain in preferred temperatures, or that their range was expanding as waters warmed. Climate-induced range shifts have long been noted in terrestrial species with restricted ranges, but have not normally been associated with an increased species coverage^2^. A link between species range and climate-induced distributional shift has not previously been reported for marine fishes, so it is unclear if restricted distributional ranges are intrinsically less common in the marine environment due to a lower level of habitat fragmentation^6^, or if distributional ranges of marine fish species are more difficult to observe and measure in the presence of continual changes in abundance^26^.

Our finding that relatively few species shifted to deeper waters as temperatures increased was at marked odds with other studies^10,13,22^, but consistent with the idea that depth shifts would only be important in regions with shallow seas or strongly stratified waters. Icelandic waters tend to be dynamic and poorly stratified in many locations and times of the year, with a continental shelf which ranges over a 1000-m depth range^27^; thus depth shifts are a less optimal means for thermoregulation for many species, especially in the southwest and northeast below a depth of 300 m.

A relationship between abundance and species range is well established, both in terrestrial and aquatic environments^28^. Our results are consistent with that paradigm, in that increased abundance led to range shifts in 62% of the species-region combinations. However, contrary to what has been reported in other marine studies^13,14^, the effect of increased abundance was less pronounced than was the effect of higher temperatures; our results indicated that a temperature increase of 1 °C would produce a 3.4 times larger geographic shift to the north than would a doubling of population abundance. Abundance changes leading to density-dependent habitat selection are frequently observed in fish populations^29^, but there is one critical characteristic that should differentiate abundance-induced shifts from climate-induced shifts; abundance-based shifts should be insensitive to orientation. While increased abundance would lead to range shifts into outlying, sub-optimal habitat in any direction, climate-induced range shifts should be oriented in line with thermal gradients (primarily poleward). In our study, we found that unoriented abundance-based shifts were of comparable magnitude to those induced by temperature increase, but that more species shifted northward, over longer distances, in response to warming. Given the much higher variance in abundance in fish populations relative to mammalian or avian populations^30^, the implications of unoriented distribution shifts resulting from population increase are a reduced ability to detect distribution shifts from climate. Although some have suggested that fishing activity by itself could shift species’ distribution^10,12,13^, we consider it more likely that any effect of fishing is through its effect on localized reductions in abundance.

The centre of mass (CoM) calculations used to quantify species-specific distribution shifts in this study do not distinguish between active directed movement of individuals versus a longer-term geographic shift in settlement or recruitment sites. Thus it is not possible to state definitively that the individuals in each population actively re-distributed to match or follow changing thermal gradients. However, the fact that *Year* was a significant factor in some models indicates that geographic shifts in population CoM must have occurred over a fairly short time frame. Detection of within-year geographic shifts between seasonal surveys would answer this question. Previous observations that small pelagic species migrate quickly to pursue thermal gradients suggests that active migration must be occurring^10,31^. Although new spawning and recruitment sites can develop given sufficient environmental change^32^, the evolutionary stability of sensitive life history sites implies that the development of new sites would not be as rapid as would active migration^18^.

The species-specific ecological attributes and analytical tools applied in this study are applicable to other marine ecosystems, and are probably more useful than the actual list of species affected. Abundance-weighted location data (such as CoM) have often been used to describe geographic re-distributions^12,13^, but have seldom been estimated in a three-dimensional linear model framework as was done here. Use of rose plots to graphically illustrate the direction and extent of movement of multiple species was a particularly useful linkage to the linear model output. In all such analyses however, the robustness of the results is dependent on the contrast in the data (range of environmental temperatures within and across surveys) and the length of the abundance time series; it would be difficult to disentangle spatial and temporal effects in very short time series, and impossible to do so in a single survey. The thermal attributes of the species, both thermal bias (i.e. warm or cold water) and tolerance (i.e. stenothermal vs eurythermal), appeared to be the most powerful predictors of climate-induced geographic shift, a conclusion also reached by studies where they were applied at the community level^11,25^. However, there is no reason to expect community structure to remain unchanged as the environment warms^33^, which argues in favour of species-specific rather than community-level thermal attributes. The quantitative measures of TB and Steno introduced in this study are easily applied to any species and thus provide a means of comparison among divergent ecosystems. Other ecological attributes which have been linked to climate-induced shifts are small pelagic species^10,31^, small bottom benthivores and large motile piscivores and generalists^22^, most or all of which would be classifed as climate-sensitive by the TB and Steno indices.

As has been noted by the IPCC for terrestrial environments^34^, it is misleading to consider marine climate warming as a homogeneous, unidirectional process. Our hydrographic station time series clearly demonstrated nonlinear but long-term warming trends in inshore stations, trends that were absent in mid-depth offshore stations. This nonlinearity also appeared in the statistical models, where the annual temperatures provided superior prediction of geographic shifts compared to that of the calendar year. Icelandic waters are known to be a dynamic oceanographic region at the interface of major southern and northern current systems^21,35^, hence it is not surprising that marine warming varied both regionally and through time. Indeed, some hydrographic stations showed no net change in temperatures after a 22-year period, underlining the fact that Icelandic marine waters have warmed relatively little over a reasonably long period of time. Nevertheless, Icelandic waters straddle the Arctic Circle, and the Arctic land masses have warmed faster than most other places on Earth^36^. Based on past trends in SST, warming patterns off Iceland and Greenland will differ appreciably from those further east in the northeast Atlantic, perhaps in part because of the deeper surrounding waters^11,18^.

The sub-polar waters around Iceland, the northeast Pacific and Antarctica represent some of the most productive and speciose coldwater environments in the world^37^. Continued warming of these marine regions seems inevitable, but the combination of oceanographic convergences and relatively deep water may well make the warming rate slower, more variable and less predictable than has been observed in other regions. Our results indicate that the home ranges of 7% of species may shift by > 100 km after as little as a 1 °C increase in water temperature, and has already resulted in the introduction of a major new Icelandic fishery for mackerel (*Scomber scombrus*), a species which was much less abundant in the region prior to warming^38^. Also expected, but not yet quantified, would be changes in the food web that accompany changes in the species composition. Indeed, rising water temperatures would increase both the metabolic rates and consumption rates of many resident species, over and above any changes in community structure. Thus a 2–3 °C warming of marine waters seems likely to produce large-scale changes in the location, composition and productivity of many fisheries, both around Iceland and in other regions of the world.

## Methods

The study area is situated in one of the most productive marine environments in the world, supporting high densities of primary and secondary producers and large groundfish fisheries^27^. The high productivity is due to Iceland’s location at the interface of warm, saline, northward-flowing Atlantic currents, and cold, low-salinity polar currents flowing south from the eastern and northern areas^21,35^. The inflow of the two water masses to the northern region is highly variable which results in dynamic changes between years and seasons. Long term monitoring of hydrographic conditions in Icelandic waters has shown a rise in temperature and salinity from 1996 with an increased inflow of warm Atlantic water to the north of Iceland^24^.

Fish distribution and ocean temperature data were based on standardized autumn groundfish surveys of the Icelandic continental shelf and slope conducted annually by the Marine and Freshwater Research Institute (MFRI) in Iceland. “Golden Top” #77 trawls were used in shallow water (0–400 m) while the larger and heavier “Golden Top” #78 trawls were used in deep water (400–1500 m). Mesh sizes were 135 mm near the front of the trawl, 80 mm in the middle section and 40 mm in the codend. Towing speed was standardized at 3.8 knots (1.95 m/s) over a trawling distance of 3.0 nautical miles (5.56 km)^39^. The surveys have been conducted annually each autumn (ranging from mid-September to early November) since 1996, and were intended to complement the annual spring surveys, especially for *Reinhardtius hippoglossoides* and *Sebastes mentella*.

The autumn survey fish collections and handling were all carried out in accordance with the guidelines, permits and regulations of the Icelandic government, as issued to the Marine and Freshwater Research Institute, Iceland.

The full autumn survey data set included about 400 fixed stations, not all of which were sampled over the entire 22-year time span. Only the subset of the stations sampled in all 22 years have been included in this analysis (Fig. 1). Thus the subset included 245 stations and 5390 sets (tows) sampled between 1996 and 2018, with the exception of 2011 when a strike prevented the survey from being carried out. Measurements recorded at each station included location, start and end tow depth and tow length. Bottom and surface temperatures were recorded with trawl temperature sensors and subsequently corrected with pre-calibrated temperature recorders^40^, although temperature measurements were not available for 3.8% of the stations. Based on oceanographic considerations, the data were sub-divided into four regions (NW, NE, SW and SE), with a dividing line between east and west Iceland at 20°W longitude, and a latitudinal division at 65.5°N in the west, and 64.5°N in the east.

The catch number of each species in each tow was standardized to the number of fish caught per nautical mile (1.85 km). An index of species-specific annual abundance (A_y_) was calculated as the within-year sum of the species’ standardized catch numbers. Differences in relative abundance among species were assessed using the mean of non-zero species-specific annual abundance across years (A_s_) for all areas or as the across-year sum of A_y_ within region (A_r_). A standardized annual index of abundance, better suited for comparisons across species, was calculated as the species-specific annual abundance divided by its mean across years (SA_y_). This within-species trend in standardized index of abundance (SA_y_) was used to test for the effect of changing abundance on distribution.

The mean spatial coverage of each species within a region was estimated using two methods. Bivariate normal kernel density estimators were calculated using the R package *adehabitatHR*^41^, whereby species coverage in each of the four regions was estimated as the contour line encompassing 90% of the estimated species distribution. Many species were distributed irregularly around the Icelandic coastline, making this approach inaccurate wherever the bivariate normal distribution assumption was violated. Thus this approach was discarded in favour of a second, more empirical approach: a simple count of unique stations occupied by each species in each region, summed across years, and not weighted by abundance (Area_sr_).

Any given fish species tends to have a specific temperature range with which it is most strongly associated^25^. The thermal bias of an individual species may be towards waters that are warmer or colder than that of the overall environment (i.e. a warmwater or coldwater species). In addition, the range of temperatures which can be tolerated or preferred can either be narrow (stenothermal) or broad (eurythermal). To characterize the thermal bias (TB) of each species, the median bottom water temperature of all stations across all years was subtracted from the median catch-weighted temperature of the species across all years. Thus a positive TB index would be indicative of a warmwater species, while negative TB indices were interpreted as coolwater (− 3 to 0) and coldwater (− 7 to − 3) species. To characterize the temperature tolerance range, the Steno index was calculated as the temperature range delimited by the 5th and 95th percentiles of the species’ catch-weighted temperatures across all years. Thus a small value for the Steno index would be indicative of a species with a very narrow temperature tolerance range. The Steno index was subsequently binned in four equal 2.5 °C ranges.

Distributional shifts of each species across years were calculated using three approaches: (1) inter-annual comparisons of the mapped standardized survey catches; (2) year-to-year shifts in the centre of mass (centroid) of the survey distribution of each species; and (3) general linear models (GLMs) predicting the latitude, longitude and depth of the survey catch as a function of species, year, temperature and abundance. Unless indicated otherwise, only the 82 species that were collected in at least 19 of the 22 years were included in any of the above analyses. However, a separate analysis was carried out for any species which first appeared (or disappeared) late in the time series, thus suggesting immigration to or from a warming environment rather than sporadic catches of a rare species. All statistical analyses were carried out in either R ver. 3.4.3 or SPSS ver. 26.

To calculate the annual centre of mass of a given species, the mean annual latitude, longitude and depth was calculated using the location of each tow, weighted by the standardized numerical abundance of the species at that location. Linear regression of the annual centre of mass against year was used as a simple indicator of long-term movement of any given species, with the regression parameters used to estimate the predicted location and depth of the species at the beginning (1996) and end (2018) of the time series. Distance and bearing between the start and end points were then calculated using the *geosphere* v1.5.10 package in R^42^. Rose plots showing the distance and bearing data by species were prepared with the *ggplot2* v3.3.0 package in R^43^.

The predicted effect of climate warming on species distribution was assessed with multivariate GLMs, first across Icelandic waters as a whole, and then in a spatially-structured analysis which allowed for directed movements within each of the four Icelandic regions that were previously defined: NW, NE, SW and SE. A spatially-structured analysis allows for directed movements of species with circum-island distributions that are geographically unable to shift their distribution in one or more directions before hitting a land mass. Latitude, longitude and depth were entered as dependent variables in the spatially-structured multivariate GLM for each species, with environmental temperature (*Temp*_*e*_, defined below) and standardized annual abundance (*SA*_*y*_) entered as covariates, and *Region* as a factor. To allow for different covariate slopes between regions, the interaction terms between *Region* and *Temp*_*e*_ and *Region* and *SA*_*y*_ were also entered into the model. For those species with significant parameter estimates for *Temp*_*e*_ either as a main effect or interaction term, distance and bearing were calculated as described earlier, and rose plots prepared for each region. Models were developed hierarchically, with model selection based on the Akaike information criterion (AIC).

### Time series of bottom water temperatures

Near-bottom water temperatures over the period 1996–2018 were available from three independent sources. Winter, spring, and autumn near-bottom temperatures (hereafter referred to as “bottom temperatures”) along two standard hydrographic sections represent areas with different hydrographic conditions. The Faxaflói section (FX) represents areas south of the Greenland–Scotland ridge characterized by Atlantic water, whereas the Siglunes section (SI) represents colder areas north of the ridge as well as the volume of Atlantic water flowing onto the northern shelf^24,44,45^. We selected three stations from each section: FX3 and SI1 (~ 70 m) represent shallow nearshore areas, FX8 and SI7 (~ 400 m, comparable to the autumn mean survey depth of 377 m) represent intermediate depths, and FX9 and SI8 (~ 1000 m) represent the deepest areas of the autumn survey. Although not representative of the entire survey area, these hydrographic stations do provide excellent time series at fixed locations.

The third source of bottom water temperatures was from the autumn trawl survey itself. A simple annual mean bottom temperature across all stations was not a valid temperature index given inter-annual variability in the timing of the survey and differences in the order of the fixed stations that were sampled. Therefore, a standardized bottom temperature index was calculated using a GLM of bottom temperature with fixed station and year as factors, and day of the year (*DOY*) as a covariate. Thus the model links the temperature to the location of each fixed station and corrects for inter-annual variation in the date that the station was fished. All terms were highly significant (*P* < 0.0001, 266 *df*) in a model explaining 94% of the variance. The slope of the *DOY* parameter was − 0.003 (SE = 0.001), implying that a 10-day difference in survey timing would only account for an inter-annual mean temperature difference of 0.03 °C. The estimated marginal mean bottom temperature resulting from the GLM was considered to be the best overall annual index of bottom temperatures integrated over the survey distribution area. In this study, it has been termed the environmental temperature (*Temp*_*e*_).

Depth-temperature profiles representative of the four regions around Iceland were calculated as mean bottom temperatures across years at each index station (fixed location and depth), then averaged within regions. Fitted lines were loess regressions.

## Supplementary information

Supplementary Informations.

## Data Availability

Data available upon request.

## References

[1] Forcada J, Trathan PN, Murphy EJ (2008). Life history buffering in Antarctic mammals and birds against changing patterns of climate and environmental variation. Glob. Change Biol..

[2] Pacifici M (2015). Assessing species vulnerability to climate change. Nat. Clim. Change.

[3] Thomas CD (2010). Climate, climate change and range boundaries. Divers. Distrib..

[4] Schloss CA, Nunez TA, Lawler JJ (2012). Dispersal will limit ability of mammals to track climate change in the Western Hemisphere. Proc. Natl. Acad. Sci..

[5] Campana SE (2020). Arctic freshwater fish productivity and colonization increase with climate warming. Nat. Clim. Change.

[6] Carr MH (2003). Comparing marine and terrestrial ecosystems: implications for the design of coastal marine reserves. Ecol. Appl..

[7] Wright PJ, Pinnegar JK, Fox C (2020). Impacts of climate change on fish, relevant to the coastal and marine environment around the UK. MCCIP Sci. Rev..

[8] Lassalle G, Rochard E (2009). Impact of twenty-first century climate change on diadromous fish spread over Europe, North Africa and the Middle East. Glob. Change Biol..

[9] Lyons J, Stewart JS, Mitro M (2010). Predicted effects of climate warming on the distribution of 50 stream fishes in Wisconsin, U.S.A. J. Fish Biol..

[10] Perry AL, Low PJ, Ellis JR, Reynolds JD (2005). Climate change and distribution shifts in marine fishes. Science.

[11] Dulvy NK (2008). Climate change and deepening of the North Sea fish assemblage: a biotic indicator of warming seas. J. Appl. Ecol..

[12] Engelhard GH, Righton DA, Pinnegar JK (2014). Climate change and fishing: a century of shifting distribution in North Sea cod. Glob. Change Biol..

[13] Adams CF (2018). Relative importance of population size, fishing pressure and temperature on the spatial distribution of nine Northwest Atlantic groundfish stocks. PLoS ONE.

[14] Last PR (2011). Long-term shifts in abundance and distribution of a temperate fish fauna: a response to climate change and fishing practices. Glob. Ecol. Biogeogr..

[15] Barbeaux SJ, Hollowed AB (2017). Ontogeny matters: climate variability and effects on fish distribution in the eastern Bering Sea. Fish. Oceanogr..

[16] Portner HO, Knust R (2007). Climate change affects marine fishes through the oxygen limitation of thermal tolerance. Science.

[17] Horodysky AZ, Cooke SJ, Brill RW (2015). Physiology in the service of fisheries science: Why thinking mechanistically matters. Rev. Fish. Biol. Fish..

[18] Brander K (2003). Changes in fish distribution in the eastern North Atlantic: Are we seeing a coherent response to changing temperature?. ICES Mar. Sci. Sympos..

[19] Brown CJ (2016). Ecological and methodological drivers of species’ distribution and phenology responses to climate change. Glob. Change Biol..

[20] MacCall AD (1990). Dynamic Geography of Marine Fish Populations.

[21] Stefansdottir L, Solmundsson J, Marteinsdottir G, Kristinsson K, Jonasson JP (2010). Groundfish species diversity and assemblage structure in Icelandic waters during recent years of warming. Fish. Oceanogr..

[22] Frainer A (2017). Climate-driven changes in functional biogeography of Arctic marine fish communities. Proc. Natl. Acad. Sci..

[23] Nawri, N. and Bjornsson, H. Surface air temperature and precipitation trends for Iceland in the 21st century. *Vedurstofa Islands Skyrsla* **VI 2010-005** (2010).

[24] Valdimarsson H, Astthorsson OS, Palsson J (2012). Hydrographic variability in Icelandic waters during recent decades and related changes in distribution of some fish species. ICES J. Mar. Sci..

[25] Burrows MT (2019). Ocean community warming responses explained by thermal affinities and temperature gradients. Nat. Clim. Change.

[26] Fisher JA, Frank KT (2004). Abundance-distribution relationships and conservation of exploited marine fishes. Mar. Ecol. Prog. Ser..

[27] Astthorsson OS, Gislason A, Jonsson S (2007). Climate variability and the Icelandic marine ecosystem. Deep-Sea Res. II.

[28] Brown JH (1984). On the relationship between abundance and distribution of species. Am. Nat..

[29] Shepherd TD, Litvak MK (2004). Density-dependent habitat selection and the ideal free distribution in marine fish spatial dynamics: considerations and cautions. Fish. Fish..

[30] Leung B, Greenberg DA, Green DM (2017). Trends in mean growth and stability in temperate vertebrate populations. Diversity Distrib..

[31] Rose GA (2005). On the distributional responses of North Atlantic fish to climate change. ICES J. Mar. Sci..

[32] Solmundsson J, Jonsson E, Bjornsson H (2010). Phase transition in recruitment and distribution of monkfish (*Lophius piscatorius*) in Icelandic waters. Mar. Biol..

[33] Beaugrand GA (2019). Prediction of unprecedented biological shifts in the global ocean. Nat. Clim. Change.

[34] Collins, M. et al. 2013: Long-term Climate Change: Projections, Commitments and Irreversibility. in *Climate Change 2013: The Physical Science Basis. Contribution of Working Group I to the Fifth Assessment Report of the Intergovernmental Panel on Climate Change* (ed. Stocker, T. F. et al.). 1029–1136 (Cambridge University Press. Cambridge, 2013).

[35] Astthorsson OS, Palsson J (2006). New fish records and records of rare southern species in Icelandic waters in the warm period 1996–2005. ICES CM.

[36] Huang J (2017). Recently amplified arctic warming has contributed to a continual global warming trend. Nat. Clim. Change.

[37] Hunt GL (2016). Advection in polar and sub-polar environments: Impacts on high latitude marine ecosystems. Prog. Oceanogr..

[38] Astthorsson OS, Valdimarsson H, Gudmundsdottir A, Óskarsson GJ (2012). Climate-related variations in the occurrence and distribution of mackerel (*Scomber scombrus*) in Icelandic waters. ICES J. Mar. Sci..

[39] Marine Research Institute. Manuals for the Icelandic bottom trawl surveys in spring and autumn. *MRI Technical Report* **156**, 125 pp (2010).

[40] Björnsson, H. et al. The Icelandic groundfish surveys in March 1985–2006 and in October 1996–2006. *Hafrannsóknir* **131**, 220 pp in Icelandic with English summary (2007).

[41] Calenge C (2006). The package adehabitat for the R software: tool for the analysis of space and habitat use by animals. Ecol. Model..

[42] Hijmans R.J. Geosphere: Spherical Trigonometry. R package version 1.5-10. https://CRAN.R-project.org/package=geosphere (2019).

[43] Wickham H. *ggplot2: Elegant Graphics for Data Analysis*. (Springer, New York, 2016). https://ggplot2.tidyverse.org. ISBN 978-3-319-24277-4.

[44] Stefansson U (1962). North Icelandic waters. Rit Fiskideildar.

[45] Valdimarsson H, Malmberg SA (1999). Near-surface circulation in Icelandic water derived from satellite tracked drifters. Rit Fiskideildar.

