# Supplementary material for: Shifting fish distributions in warming sub-Arctic oceans
Source: Sci Rep. 2020 Oct 5;10:16448. doi: 10.1038/s41598-020-73444-y (PMC7536214; doi:10.1038/s41598-020-73444-y)

Supplementary Information for
Shifting fish distributions in warming sub-Arctic oceans

Steven E. Campana[1*], Ragnhildur B. Stefánsdóttir[2], Klara Jakobsdóttir[3] and Jón Sólmundsson[4]

[1] Life and Environmental Science, University of Iceland, 101 Reykjavik, Iceland [354-840-2802]; e-mail *scampana@hi.is*

[2] Life and Environmental Science, University of Iceland, 101 Reykjavik, Iceland; e-mail *rbs4@hi.is*

[3] Marine and Freshwater Research Institute, Hafnarfjörður, Iceland; e-mail klara.jakobsdottir@hafogvatn.is

[4] Marine and Freshwater Research Institute, Hafnarfjörður, Iceland; e-mail jon.solmundsson@hafogvatn.is

Supplementary Table 1. Summary table of 82 fish species captured in more than 18 annual autumn surveys around Iceland.

Species code	Species	Mean depth (m)	Mean temperature	Steno index	Thermal bias index	Mean annual survey abundance
1	Gadus morhua	242	3.23	7.98	-2.45	3633
2	Melanogrammus aeglefinus	170	7.51	4.69	2.20	19541
3	Pollachius virens	213	7.13	5.29	1.46	648
4	Merlangius merlangus	143	8.82	2.93	3.20	2182
5	Sebastes marinus	228	7.12	2.13	1.60	17068
6	Molva molva	193	7.65	2.30	2.00	28
7	Molva dypterygia	492	6.56	5.15	1.44	128
8	Brosme brosme	292	5.85	8.13	1.17	31
9	Anarhichas lupus	177	5.83	6.25	0.53	366
10	Coryphaenoides rupestris	935	4.32	1.46	-1.31	4753
12	Amblyraja radiata	309	2.83	9.20	-3.36	363
13	Anarhichas minor	305	3.50	8.40	-2.03	48
14	Lophius piscatorius	180	8.07	2.84	2.40	5
16	Squalus acanthias	130	8.40	3.23	2.86	3
19	Argentina silus	367	6.94	3.79	1.50	2662
21	Hippoglossus hippoglossus	136	8.53	4.16	2.90	7
22	Reinhardtius hippoglossoides	647	0.44	5.92	-5.94	793
23	Pleuronectes platessa	111	8.34	4.10	2.66	359
24	Microstomus kitt	149	8.37	3.09	2.76	431
25	Glyptocephalus cynoglossus	182	7.88	3.50	2.53	234
26	Lepidorhombus whiffiagonis	191	8.16	2.01	2.58	39
27	Limanda limanda	102	8.61	2.31	2.88	1479
28	Hippoglossoides platessoides	234	6.02	8.27	0.95	4035
30	Clupea harengus	180	7.53	4.86	2.00	1859
33	Trisopterus esmarkii	180	7.59	2.24	1.90	27513
34	Micromesistius poutassou	381	6.78	5.32	1.60	15343
39	Chimaera monstrosa	378	7.48	1.91	1.97	36
47	Anarhichas denticulatus	523	2.96	7.28	-2.40	8
48	Cyclopterus lumpus	198	4.90	6.06	-0.50	19
49	Hoplostethus atlanticus	1007	4.12	0.82	-1.43	9
53	Triglops murrayi	204	4.35	8.75	-0.12	40
56	Leptagonus decagonus	465	-0.24	3.22	-5.97	22
57	Rhinonemus cimbrius	181	7.48	4.17	2.10	11
58	Lepidion eques	771	5.53	3.49	-0.20	48
59	Lycodes reticulatus	469	-0.35	2.01	-5.95	22
60	Sebastes viviparus	250	7.63	1.94	2.00	6619
61	Sebastes mentella	503	5.62	4.21	0.40	2178
62	Macrourus berglax	870	2.70	5.85	-1.58	20
63	Lycodes esmarkii	512	0.34	6.65	-5.90	37
64	Etmopterus princeps	874	4.49	2.81	-1.24	25
65	Leptoclinus maculatus	208	5.26	8.84	0.44	1
67	Gadiculus argenteus thori	337	7.63	0.93	2.06	15
69	Lycodes seminudus	615	-0.37	2.21	-6.06	59
70	Careproctus reinhardti	580	-0.46	2.13	-6.06	85
71	Boreogadus saida	395	2.29	7.90	-5.36	93
74	Artediellus atlanticus	371	0.26	6.10	-5.83	117
79	Lycodes gracilis	255	2.76	7.47	-2.29	44
81	Cottunculus microps	575	0.18	6.54	-5.99	14
82	Bathyraja spinicauda	639	2.48	5.74	-2.41	1
87	Rajella fyllae	602	3.36	7.55	-1.10	8
88	Gaidropsarus argentatus	622	-0.02	5.91	-6.01	20
90	Amblyraja hyperborea	691	-0.56	1.58	-6.09	17
94	Lumpenus lampretaeformis	165	7.02	4.36	1.44	5
96	Centroscyllium fabricii	875	4.50	2.40	-1.20	187
98	Lycodes eudipleurostictus	610	-0.55	1.50	-6.08	125
99	Eutrigla gurnardus	137	8.44	1.67	2.80	75
104	Xenodermichthys copei	739	3.72	6.10	-0.93	2
105	Notacanthus chemnitzii	889	3.82	4.97	-1.39	34
106	Borostomias antarcticus	1006	4.36	1.65	-1.26	1
107	Synaphobranchus kaupii	968	4.21	3.80	-1.25	25
110	Liparis fabricii	652	-0.60	1.60	-6.14	3
113	Lycodes pallidus	612	-0.39	1.96	-5.98	42
114	Apristurus laurussonii	1010	4.40	1.44	-1.24	7
117	Coryphaenoides guentheri	1014	4.43	1.58	-1.23	27
118	Galeus murinus	835	5.32	3.09	-0.40	7
123	Arctozenus risso	740	3.27	6.80	-1.34	5
125	Etmopterus spinax	473	7.04	2.09	1.46	32
136	Bathylagus euryops	1003	3.89	4.51	-1.39	14
150	Helicolenus dactylopterus	260	7.71	1.89	2.05	42
153	Cottunculus thomsonii	873	3.72	6.71	-1.40	1
154	Serrivomer beanii	932	4.34	3.41	-1.20	5
161	Alepocephalus bairdii	990	4.49	1.50	-1.23	227
164	Centroscymnus crepidater	781	5.63	3.38	0.34	5
169	Harriotta raleighana	988	4.41	1.53	-1.17	1
171	Trachyrincus murrayi	1005	4.42	1.50	-1.23	82
173	Aphanopus carbo	904	5.10	2.21	-0.57	47
174	Centroscymnus coelolepis	912	4.75	2.95	-0.98	4
175	Rhinochimaera atlantica	990	4.41	1.69	-1.25	6
207	Scopelosaurus lepidus	1009	4.46	1.50	-1.20	7
216	Lycodes squamiventer	718	0.16	5.13	-5.99	3
248	Magnisudis atlantica	905	4.61	2.75	-1.10	21
301	Ammodytes marinus	117	8.61	4.20	2.86	37

Suppl. Table 2a. Summary results from GLM of fish temperature at capture as a function of *Species* (r^2=0.51)

Source	Sum of Squares	df	Mean Square	F	Sig.	Power
Corrected Model	4299880	81	53084.9	30450.4	0.000	1.000
Intercept	146794	1	146793.7	84203.4	0.000	1.000
Species	4299880	81	53084.9	30450.4	0.000	1.000
Error	4181505	2398582	1.7			
Total	123008241	2398664				
Corrected Total	8481385	2398663				

Suppl. Table 2b. Summary results from GLM of fish temperature at capture as a function of *Species* with *Year* as a covariate (r^2=0.51).

Source	Sum of Squares	df	Mean Square	F	Sig.	Power
Corrected Model	4300474	82	52444.8	30087.5	0.000	1.000
Intercept	7	1	7.3	4.2	0.040	0.536
Year	594	1	594.1	340.8	0.000	1.000
Species	4278425	81	52820.1	30302.8	0.000	1.000
Error	4180911	2398581	1.7			
Total	123008241	2398664				
Corrected Total	8481385	2398663				

Suppl. Table 2c. Summary results from GLM of fish temperature at capture as a function of *Species* with $Temp_e$ (environmental temperature) and *Depth* as covariates (r^2=0.62).

Source	Sum of Squares	df	Mean Square	F	Sig.	Power
Corrected Model	5231383	83	63028.7	46516.7	0.000	1.000
Intercept	83756	1	83755.8	61813.8	0.000	1.000
$Temp_e$	62071	1	62071.4	45810.2	0.000	1.000
Depth	844845	1	844844.6	623515.9	0.000	1.000
Species	2601312	81	32115.0	23701.6	0.000	1.000
Error	3250001	2398580	1.4			
Total	123008241	2398664				
Corrected Total	8481385	2398663				

Suppl. Table 2d. Summary results from GLM of fish temperature at capture as a function of *Species* with $Temp_e$ (environmental temperature), *Depth* and SA_y (species' standardized annual abundance) as covariates, plus the interaction terms between *Species* and SA_y (r^2=0.62).

Source	Sum of Squares	df	Mean Square	F	Sig.	Power
Corrected Model	5255873	165	31853.8	23686.5	0.000	1.000
Intercept	19123	1	19122.8	14219.7	0.000	1.000
Species	363883	81	4492.4	3340.5	0.000	1.000
Depth	827824	1	827824.5	615572.3	0.000	1.000
$Temp_e$	59433	1	59432.7	44194.3	0.000	1.000
SA_y	5	1	5.2	3.9	0.048	0.506
Species * Sa_y	23384	81	288.7	214.7	0.000	1.000
Error	3225511	2398498	1.3			
Total	123008241	2398664				
Corrected Total	8481385	2398663				

Suppl. Table 3. Summary results from GLM of fish depth at capture as a function of *Species* with *Year* as a covariate (r^2=0.88).

Source	Sum of Squares	df	Mean Square	F	Sig.	Power
Corrected Model	1.02E+11	1635	62471460.2	10689.7	0.000	1.000
Intercept	1.59E+09	1	1585018691.0	271217.0	0.000	1.000
Species	8.29E+10	81	1023005122.7	175049.3	0.000	1.000
Year	6.98E+05	21	33245.5	5.7	0.000	1.000
Species * Year	1.39E+09	1533	908231.0	155.4	0.000	1.000
Error	1.47E+10	2507404	5844.1			
Total	2.70E+11	2509040				
Corrected Total	1.17E+11	2509039				

Suppl. Table 4a. Summary results from multivariate GLM of latitude, longitude and depth at capture as a function of *Species* with *Year* and SA_y (species' standardized annual abundance) as covariates. R^2 = 0.228 (Lat), 0.319 (Long), 0.861 (Depth).

Source	Dependent variable	Sum of Squares	df	Mean Square	F	Sig.	Power
Corrected Model	lat	2287921	83	27565.3	26803.0	0.000	1.000
	lon_pos	48573261	83	585220.0	42618.5	0.000	1.000
	depth	302453065879	83	3644012841.9	560417.0	0.000	1.000
Intercept	lat	122105	1	122105.3	118728.5	0.000	1.000
	lon_pos	160237	1	160237.1	11669.2	0.000	1.000
	depth	4364296	1	4364295.6	671.2	0.000	1.000
Year	lat	31696	1	31696.0	30819.5	0.000	1.000
	lon_pos	331178	1	331178.5	24118.0	0.000	1.000
	depth	5117140	1	5117140.1	787.0	0.000	1.000
Say	lat	742	1	742.1	721.6	0.000	1.000
	lon_pos	501248	1	501247.6	36503.2	0.000	1.000
	depth	862316394	1	862316393.6	132616.7	0.000	1.000
Species	lat	2274306	81	28077.8	27301.4	0.000	1.000
	lon_pos	45985513	81	567722.4	41344.3	0.000	1.000
	depth	297488589356	81	3672698634.0	564828.7	0.000	1.000
Error	lat	7750660	7536318	1.0			
	lon_pos	103485642	7536318	13.7			
	depth	49003576348	7536318	6502.3			
Total	lat	31505332942	7536402				
	lon_pos	3901373181	7536402				
	depth	811197402105	7536402				
Corrected Total	lat	10038581	7536401				
	lon_pos	152058903	7536401				
	depth	351456642227	7536401				

Suppl. Table 4b. Summary results from multivariate GLM of latitude, longitude and depth at capture as a function of *Species* with *Year* and SA_y (species' standardized annual abundance) as covariates, plus their interaction terms with *Species*. R^2 = 0.259 (Lat), 0.342 (Long), 0.864 (Depth).

Source	Dependent variable	Sum of Squares	df	Mean Square	F	Sig.	Power
Corrected Model	Lat	2595536	236	10998	11135.6	0.000	1.000
	Long	52074219	236	220653	16631.4	0.000	1.000
	Depth	303709587163	236	1286905030	203118.9	0.000	1.000
Species	Lat	107765	80	1347	1363.9	0.000	1.000
	Long	743823	80	9298	700.8	0.000	1.000
	Depth	277673939	80	3470924	547.8	0.000	1.000
Year	Lat	39	1	39	39.2	0.000	1.000
	Long	1786	1	1786	134.6	0.000	1.000
	Depth	433	1	433	0.1	0.794	0.058
Species * Year	Lat	108010	80	1350	1367.0	0.000	1.000
	Long	721049	80	9013	679.3	0.000	1.000
	Depth	245860669	80	3073258	485.1	0.000	1.000
Say* Species	Lat	149362	74	2018	2043.7	0.000	1.000
	Long	1375813	74	18592	1401.3	0.000	1.000
	Depth	685597658	74	9264833	1462.3	0.000	1.000
Error	Lat	7443046	7536165	1			
	Long	99984684	7536165	13			
	Depth	47747055064	7536165	6336			
Total	Lat	31505332942	7536402				
	Long	3901373181	7536402				
	Depth	811197402105	7536402				
Corrected Total	Lat	10038581	7536401				
	Long	152058903	7536401				
	Depth	351456642227	7536401				

Suppl. Table 5. Summary results from multivariate GLM of latitude, longitude and depth at capture as a function of *Species* with $Temp_e$ (environmental temperature) and SA_y (species' standardized annual abundance) as covariates, plus their interaction terms with *Species*. R^2 = 0.254 (Lat), 0.34 (Long), 0.86 (Depth)

Source	Dependent variable	Sum of Squares	df	Mean Square	F	Sig.	Power
Corrected Model	Lat	2552265	245	10417	10485.0	0.000	1.000
	Long	51418200	245	209870	15711.3	0.000	1.000
	Depth	303729259178	245	1239711262	195165.3	0.000	1.000
Intercept	Lat	553167	1	553167	556757.9	0.000	1.000
	Long	54039	1	54039	4045.5	0.000	1.000
	Depth	47850218	1	47850218	7533.0	0.000	1.000
Species	Lat	49348	81	609	613.2	0.000	1.000
	Long	427758	81	5281	395.3	0.000	1.000
	Depth	942212157	81	11632249	1831.2	0.000	1.000
Tempe	Lat	22	1	22	21.9	0.000	0.997
	Long	463	1	463	34.7	0.000	1.000
	Depth	769727	1	769727	121.2	0.000	1.000
Say	Lat	11	1	11	11.5	0.001	0.925
	Long	135	1	135	10.1	0.001	0.889
	Depth	1260113	1	1260113	198.4	0.000	1.000
Species*Tempe	Lat	57252	81	707	711.4	0.000	1.000
	Long	217730	81	2688	201.2	0.000	1.000
	Depth	137296930	81	1695024	266.8	0.000	1.000
Say* Species	Lat	205850	81	2541	2557.9	0.000	1.000
	Long	2329708	81	28762	2153.2	0.000	1.000
	Depth	960762033	81	11861260	1867.3	0.000	1.000
Error	Lat	7488427	7537041	1			
	Long	100679290	7537041	13			
	Depth	47876110983	7537041	6352			
Total	Lat	31509126562	7537287				
	Long	3901781282	7537287				
	Depth	811515712134	7537287				
Corrected Total	Lat	10040692	7537286				
	Long	152097490	7537286				
	Depth	351605370161	7537286				

Suppl. Table 6. Summary results from multivariate GLM of latitude, longitude and depth at capture as a function of *Region* with $Temp_e$ (environmental temperature) and SA_y (species' standardized annual abundance) as covariates, plus their interaction terms with *Region* R^2 = 0.40 (Lat),0.61 (Long), 0.03 (Depth). The model was fit separately for each species; the output example is that of cod (*Gadus morhua*).

Source	Dependent variable	Sum of Squares	df	Mean Square	F	S
Corrected Model	lat	87774	11	7979.5	14423.1	
	lon_pos	2875065	11	261369.6	34011.5	
	depth	65752220	11	5977474.5	674.3	
Intercept	lat	626660	1	626660.4	1132700.6	
	lon_pos	72958	1	72958.0	9493.9	
	depth	5072622	1	5072622.3	572.2	
Region	lat	644	3	214.6	387.9	
	lon_pos	847	3	282.4	36.7	
	depth	6890060	3	2296686.8	259.1	
$Temp_e$	lat	4	1	4.3	7.7	
	lon_pos	1610	1	1610.4	209.6	
	depth	275000	1	275000.1	31.0	
SA_y	lat	0	1	0.0	0.0	
	lon_pos	677	1	677.4	88.1	
	depth	4907	1	4907.3	0.6	
Region * $Temp_e$	lat	284	3	94.7	171.2	
	lon_pos	2768	3	922.5	120.0	
	depth	6280936	3	2093645.5	236.2	
Region * SA_y	lat	55	3	18.4	33.3	
	lon_pos	2308	3	769.4	100.1	
	depth	5577854	3	1859284.6	209.7	
Error	lat	132647	239762	0.6		
	lon_pos	1842507	239762	7.7		
	depth	2125454200	239762	8864.9		
Total	lat	1050656989	239774			
	lon_pos	72712689	239774			
	depth	17478100558	239774			
Corrected Total	lat	220421	239773			
	lon_pos	4717573	239773			
	depth	2191206420	239773			

Source	Dependent variable	Sum of Squares	df	Mean Square	F	S
Corrected Model	lat	87774	11	7979.5	14423.1	
	lon_pos	2875065	11	261369.6	34011.5	
	depth	65752220	11	5977474.5	674.3	
Intercept	lat	626660	1	626660.4	1132700.6	
	lon_pos	72958	1	72958.0	9493.9	
	depth	5072622	1	5072622.3	572.2	
Region	lat	644	3	214.6	387.9	
	lon_pos	847	3	282.4	36.7	
	depth	6890060	3	2296686.8	259.1	
$Temp_e$	lat	4	1	4.3	7.7	
	lon_pos	1610	1	1610.4	209.6	
	depth	275000	1	275000.1	31.0	
SA_y	lat	0	1	0.0	0.0	
	lon_pos	677	1	677.4	88.1	
	depth	4907	1	4907.3	0.6	
Region * $Temp_e$	lat	284	3	94.7	171.2	
	lon_pos	2768	3	922.5	120.0	
	depth	6280936	3	2093645.5	236.2	
Region * SA_y	lat	55	3	18.4	33.3	
	lon_pos	2308	3	769.4	100.1	
	depth	5577854	3	1859284.6	209.7	
Error	lat	132647	239762	0.6		
	lon_pos	1842507	239762	7.7		
	depth	2125454200	239762	8864.9		
Total	lat	1050656989	239774			
	lon_pos	72712689	239774			
	depth	17478100558	239774			
Corrected Total	lat	220421	239773			
	lon_pos	4717573	239773			
	depth	2191206420	239773			

Suppl. Table 7. Physiological and population predictors of the distance moved by fish species in four regions around Iceland in response to a 1° increase in water temperature. The 67 species entered into the model were those that showed significant temperature responses in the spatially-explicit model described in the text. The *Steno*, *TB* and $Area_{sr}$ (Species Coverage) indices were each entered as covariates, while a three-level *Depth* bin was entered as a factor. Regional species' abundance (A_r) and its interaction terms were all non-significant in a more complete model, and thus excluded as terms here. R^2 = 0.365.

Source	Sum of Squares	df	Mean Square	F	Sig.	Power
Corrected Model	354499	25	14180	10.6	0.000	1.000
Intercept	72779	1	72779	54.3	0.000	1.000
Region	59066	3	19689	14.7	0.000	1.000
Depth	49805	2	24903	18.6	0.000	1.000
Steno	6	1	6	0.0	0.949	0.050
TB	5048	1	5048	3.8	0.053	0.491
$Area_{sr}$	57056	1	57056	42.6	0.000	1.000
Regio*Depth	85741	6	14290	10.7	0.000	1.000
Depth*Steno	43006	2	21503	16.0	0.000	1.000
Depth*TB	21391	2	10695	8.0	0.000	0.955
Region*Steno	29928	3	9976	7.4	0.000	0.985
Region*TB	18585	3	6195	4.6	0.003	0.890
Steno*TB	14902	1	14902	11.1	0.001	0.914
Error	523960	391	1340			
Total	1972275	417				
Corrected Total	878459	416				

Supplementary Fig. 1. Distribution of bottom temperatures by depth and year in the four regions around Iceland.

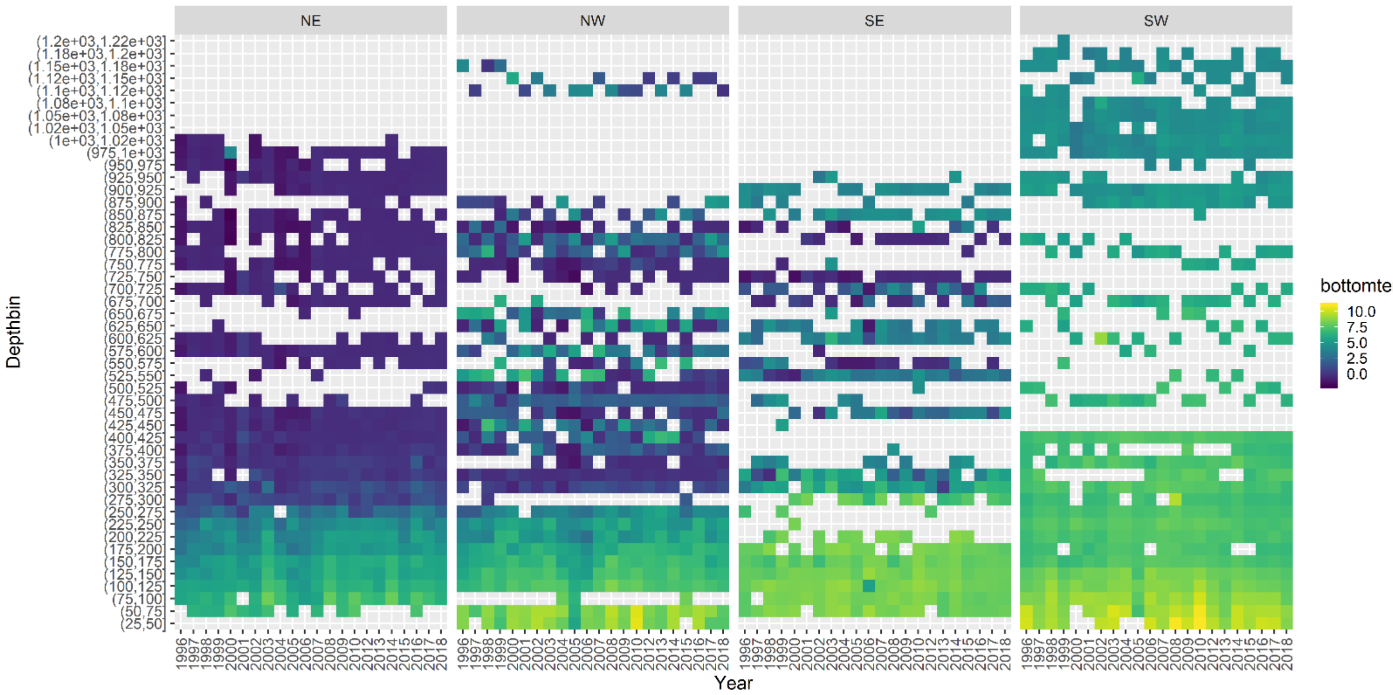

Supplementary Fig. 2. Survey catch locations of grey gurnard, *Eutrigla gurnardus*, at 5-yr intervals between 1996 and 2018 in the autumn survey. Symbol size is proportional to catch number.

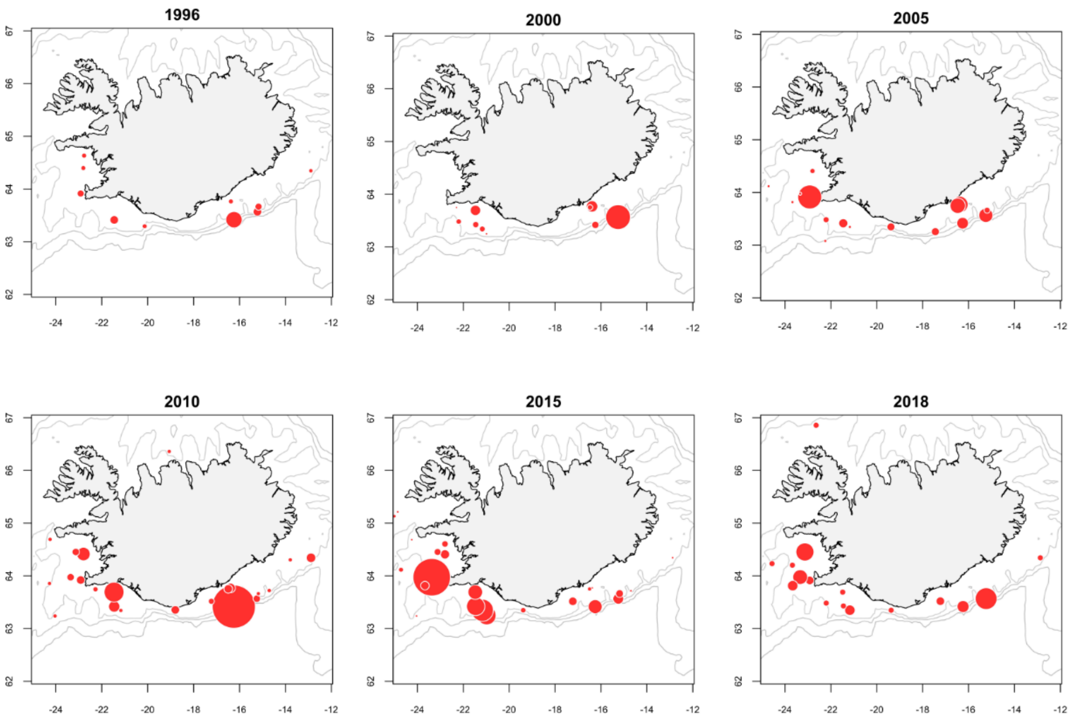

Supplement: Supplementary file 1 — Supplementary Informations. [file 41598_2020_73444_MOESM1_ESM.pdf]
